# Shaping the Future of Rare Diseases after a Global Health Emergency: Organisational Points to Consider

**DOI:** 10.3390/ijerph17228694

**Published:** 2020-11-23

**Authors:** Rosaria Talarico, Diana Marinello, Sara Cannizzo, Andrea Gaglioti, Simone Ticciati, Claudio Carta, Yllka Kodra, Mojgan Azadegan, Domenica Taruscio, Marta Mosca, Giuseppe Turchetti

**Affiliations:** 1 Rheumatology Unit, Azienda Ospedaliero Universitaria Pisana Rheumatology Unit, University of Pisa, 56126 Pisa, Italy; sara.talarico76@gmail.com (R.T.); diana.marinello@gmail.com (D.M.); sara.cannizzo@santannapisa.it (S.C.); gaglio1975@gmail.com (A.G.); ticciatisimone@gmail.com (S.T.); marta.mosca@med.unipi.it (M.M.); 2 Institute of Management, Scuola Superiore Sant’Anna, 56127 Pisa, Italy; 3 National Centre for Rare Diseases, Istituto Superiore di Sanità, 00161 Rome, Italy; claudio.carta@iss.it (C.C.); yllka.kodra@iss.it (Y.K.); domenica.taruscio@iss.it (D.T.); 4 Tuscan Regional Center for Gender Specific Medicine, Azienda Ospedaliero Universitaria Pisana, 56126 Pisa, Italy; m.azadegan@ao-pisa.toscana.it; 5 Rheumatology Unit, University of Pisa, 56126 Pisa, Italy

**Keywords:** rare diseases, COVID-19, healthcare organization, organizational models, health emergencies, health policies, rare diseases networks

## Abstract

The unexpected outbreak of the COVID-19 disease had significant and enormous repercussions on the healthcare systems, such as the need to reorganise healthcare organisations in order to concentrate resources needed to the care of COVID-19 patients and to respond in general to this health emergency. Due to these challenges, the care of several chronic conditions was in many cases discontinued and patients and healthcare professionals treating these conditions had to cope with this new scenario. This was the case of the world rare diseases (RDs) that had to face this global emergency despite the vulnerability of people with RDs and the well-known need for high expertise required to treat and manage them. The numerous lessons learned so far regarding health emergencies and RDs should represent the basis for the establishment of new healthcare policies and plans aimed at ensuring the preparedness of our health systems in providing appropriate care to people living with RDs in the case of eventual new emergencies. This paper aims at providing pragmatic considerations that might be useful in designing future actions to create or optimise existing organisational models for the care of RDs in case of future emergencies or any other situation that might threaten the provision of routine care. These policies and plans should benefit from the multi-stakeholder RDs networks (such as the European Reference Networks), that should join forces at European, national, and local levels to minimise the economic, organisational, and health-related impact and the negative effects of potential emergencies on the RDs community. In order to design and develop these policies and plans, a decalogue of points to consider were developed to ensure appropriate care for people living with RDs in the case of eventual future health emergencies.

## 1. Introduction

Public health emergencies are usually defined in relation to the consequences they generate for the health of the population and to their precipitating events. When a situation has the potential of overwhelming health routine capabilities to address a specific condition, that situation can be considered as a public health emergency [1], and this is exactly what has recently happened with the COVID-19 pandemic [2]. The COVID-19 disease is an infectious disease caused by the new coronavirus that was first identified at the beginning of 2020 in Wuhan, China. Due to the infectivity and the high contagion rate across the world, the World Health Organization (WHO) declared the COVID-19 a global pandemic on 11 March 2020. The main symptoms related to the SARS-CoV-2 infection are flu-like symptoms, severe lung injury, and, in high-risk individuals, the complications can include acute respiratory distress syndrome and multiorgan failure. As reported by the WHO, around 80% of cases are usually mild, while around 15% of people react severely and another 5% can become critically unwell. The unprecedented number of hospitalisations and often of intensive care beds, high-level of respiratory support, and personal protective equipment needed to provide appropriate care are only a few of the major difficulties that healthcare systems have experienced during the pandemic. The unexpected outbreak of the COVID-19 had, in fact, significant repercussions on the healthcare systems, such as the need to reorganise the systems in order to concentrate resources needed to the care of COVID-19 patients and to respond to this health emergency. Due to these challenges, the care of several chronic conditions was in many cases discontinued and patients and healthcare professionals treating these conditions had to cope with this new scenario. Rare diseases (RDs) often impact patients and their families by limiting their activities and affect societies implying by healthcare costs, work loss, disability pensions, early retirement, and the increasing need for social support. In addition, they carry an important burden of morbidity and mortality. Therefore, it is easy to understand how COVID-19 had and has enormous repercussions on people living with rare diseases. The results of a recent survey [3] conducted by The European Organisation for Rare Diseases (EURORDIS) has provided a valuable overview of how the COVID-19 health emergency has impacted on RDs communities and highlighted that the main challenges were the interruption of care and of the rehabilitation therapies. More than 5000 rare disease patients and families from all over Europe have answered this survey during the pandemic (from 18 to 28 April 2020), providing important results and representing around 993 rare diseases. These results have reinforced the awareness that the vulnerability of people living with RDs requires from healthcare systems a growing commitment to increase their preparedness in the case of future health emergencies and to ensure that appropriate actions are taken to make sure that people living with RDs are not left behind [4,5,6]. This paper aims at providing pragmatic considerations that might be useful in designing future actions to create or optimise existing organisational models for the care of RDs in case of future emergencies or any other situation that might threaten the provision of routine care.

## 2. The Vulnerability of RDs

In the EU, a disease is considered rare when affecting fewer than 5 people in 10,000 and although this might appear small, it translates into approximately 246,000 people. Moreover, many patients suffer from very rare diseases, as they affect one person in 100,000 or more. Approximately 5000–8000 distinct RDs affect 6–8% of the EU population, which means between 27 and 36 million people and around 400 million people worldwide. RDs are serious and chronic diseases, and in many cases life-threatening.

For the majority of RDs, there is still a very limited amount of information and of evidence available on the epidemiology of a wide number of RDs, especially regarding the prevalence and the incidence of those diseases at national and global levels. RD challenges are substantially different from the more common diseases and the major challenges are represented by the small number of patients and the consequent lack of knowledge about the many aspects of the diseases, such as the natural course and the cause of the diseases or the availability of targeted drugs for RDs. Moreover, due to their complexity and the risk of complications, they usually require a complex multidisciplinary approach that involves different experts from different disciplines. The scarce availability of expertise centres and of expert clinicians trained for the treatment and management of RDs do not allow the provision of a homogenous care and access to therapies for RD patients across the different countries.

In the last decades, many efforts were dedicated to address the RDs challenges. Among those efforts, the most promising initiative seems to be the establishment of the European Reference Networks (ERNs), virtual networks of centres of expertise for rare and complex diseases that collaborate to improve the lives of patients. The ERNs were established in 2017 by the European Commission in the framework of the Directive 2011/24/EU on patients’ rights in cross-border healthcare. Currently, 24 ERNs are actively working with patient representative (the European Patients Advocacy Groups) on 24 groups of RDs, ranging from rare metabolic diseases to rare connective tissue diseases and rare tumours. ERNs are developing Clinical Practice Guidelines, patients’ pathways, European-wide RD registries, and different educational activities (webinars, Masters, etc.) both for healthcare professionals and patients. In addition, ERNs are providing their expertise in virtual consultations thanks to the Clinical Patient Management System (CPMS), an on-line based, secure platform where clinicians and researchers across Europe convene to discuss RD clinical cases.

With regards to research, the International Rare Diseases Research Consortium (IRDiRC) and the European Joint Programme on Rare Diseases (EJP-RD) are interacting and cooperating with ERNs and other relevant stakeholders to advance the level of knowledge related to RDs management.

## 3. How an Emergency Can Impact the Organizational System of RDs Care: The Example of COVID-19

During the COVID-19 outbreak, healthcare systems had to reorganise themselves in order to respond to this global pandemic at different levels. One of the main challenges was the reorganisation of care needed to care for COVID-19 patients and the consequent lack of resources in non-COVID-19 departments/units. Many of these departments/units were taking care of RDs patients and, in different cases, the majority of their activities were temporarily suspended to be dedicated to COVID-19 care, due to the fact that many healthcare professionals had to serve in the COVID units and therefore those units were not always able to provide full care to their RDs patients. This resulted, in many cases, in a discontinuation, interruption or delay of care for RDs patients, and in the need to reorganize the patients’ flow for in- and out-patient clinics and of the related protocols as soon as the emergency decreased in severity to protect patients from the risk of infection as well as to respect the social/physical distancing rules. During the emergency, many RDs patients were not able to access to their healthcare provider, many of them could not perform the monitoring exams needed for their disease, or could not complete their diagnosis pathway, or, sometimes, the needed interventions (surgical or treatment-based) could not be organised. The implications of these limitations will have to be assessed once the health emergency will be resolved, but it is possible to hypothesize that the possible results of the discontinuation of care might include exacerbation of the disease activity, delay in diagnosis and treatment with potential accrual of organ damage, and a lack of prevention protocols. That is not to mention the psychological implications that the discontinuation of care might have had on patients, including depression, worsening of the symptoms, anxiety, isolation, fear of contracting the virus in hospitals, etc.

One of the proposed solutions to the discontinuation of care was telemedicine. During the pandemic, in fact, telemedicine and eHealth were often used for virtual consultation (e.g., phone, video o email-based consultation) as possible alternatives to face-to-face consultations. Overall, the sudden spread of the use of telemedicine and eHealth has demonstrated that it is actually possible to use this kind of tool in appropriate situations. However, it has also showed that both patients and healthcare providers are not yet fully prepared or equipped to adopt these solutions and that there is a high need to provide harmonised, user-friendly, and sustainable eHealth and telemedicine tools.

Although there is a growing number of researches aimed at assessing the impact of the pandemic on different disease populations, low evidence is still available on the RDs communities with respect to the COVID-19 outbreak and its repercussions of these communities. Recently, a cross-sectional study from China tried to examine the impact of COVID-19 pandemic on the RDs population in Hong Kong, a region with the highest number of COVID-19 confirmed cases in China outside of Hubei [7]. The study was conducted between 10 April and 29 April 2020, around 2–4 weeks after the establishment of the public gathering restrictions in Hong Kong; patients with RDs or their caregivers were recruited online through Rare Disease Hong Kong (RDHK), a big RD patient group, and other online social media platforms on a strictly voluntary basis. A structured online quantitative survey comprising of 37 questions was distributed to the participants through internet-based sources. The survey included questions regarding the patient’s demographic characteristics, health status, service use patterns, reasons for reduced service use, daily living and social life, and financial status from the patient or caregiver perspective. Participants were also asked to complete the Barthel Index for Activities of Daily Living (ADL) for the patient, which measures a person’s ability to perform ten basic activities of daily living and provides a quantitative estimate of the subject’s level of dependence. The study collected a total of 272 responses, of which 63% was from RDs patients and 38% from caregivers of patients with RDs. Even if there were no reported COVID-19 cases in the study population, the majority of patients claimed that the care was highly affected by the COVID-19 pandemic. In fact, the results of the study clearly demonstrated the patient’s and caregivers’ perception of the pandemic’s impact on RDs patient’s health and social well-being in two different dimensions: the effect brought by the closure/disruption of the public healthcare services and the perceived risk of infection. These considerations highlight how important it is to assess the impact of this pandemic in the RDs population, since we can easily imagine that all the people around the world felt more vulnerable, but RDs patients were definitely even more vulnerable than usual.

## 4. Resilience and Strategy Plans

Despite the many challenges faced during the COVID-19 pandemic, the RDs communities have shown their consistent resilience at different levels. Several initiatives and actions were promptly put in place to respond to the outbreak of the virus and to the needs of the RDs communities.

RDs Patient’s Organisations and Federations have engaged in a considerable number of new activities, joining forces at national, European, and international level to support their communities in this difficult period. A few examples include the organisation of national and European surveys aimed at identifying the main challenges experienced by people living with RDs, the tangible actions taken to safeguard the rights and the needs of people with RDs during the pandemic and, of course, the tremendous support given to these communities by their respective associations with the organisation of useful virtual initiatives.

Many RDs institutions and other initiatives have also provided a prompt response to the COVID-19 outbreak. The European Commission has promoted different actions, such as the implementation of a virtual platform (similar to the platform already used by the European Reference Networks (ERNs), the Clinical Patient Management System) to exchange information on treatment and management of COVID-19 as well as to discuss clinical cases and improve training. In addition, a series of training webinars have been organised on COVID-19 and rare and complex diseases with the support of ERNs [8]. ERNs are also contributing to support the RDs communities in different ways, developing useful considerations regarding the correlation of some RDs with COVID-19, besides other communication and educational activities [9,10].

In addition, existing Networks and scientific societies have also promptly joined forces to develop or implement patient’s registries with the aim of collecting evidence on the potential correlation of COVID-19 with RDs and other diseases, such as the Share4Rare registry that was recently built to collect evidence on people living with a RD or without a defined diagnosis and who have been confirmed to have a SARS-CoV-2 infection [11].

In Italy, another example of RD initiative is also represented by the Italian National Rare Diseases Centre (CNMR) [12], formally established within the National Institute of Health (Istituto Superiore di Sanità), that has established a task force dedicated to RDs and COVID-19, that involves experts in RDs. During the pandemic, the task force has developed several ad interim guidance documents [13] that aim at providing scientific information to support the management of several RDs during the SARS-CoV-2 emergency (e.g., acute multisystem inflammatory syndrome in children and adolescents). In addition, the CNMR has also organized regular weekly webinars (from April to July) open to the general public, not only to discuss and update the community on the status of the pandemic, but also on specific aspects (diagnosis, treatment, follow-up) of RDs in relation to COVID-19, involving RDs experts, ERN coordinators, WHO and European Commission representatives, policy makers, patients’ organisations, and other stakeholders.

All these initiatives clearly demonstrate how the RDs community acts as a wide Network to support their vulnerable members even during a global pandemic. However, such initiatives should be further implemented in the future, following this approach also at local level to ensure that healthcare systems will take into consideration people living with RDs also during future emergencies in the best way possible.

## 5. Organisational Points to Consider to Ensure Appropriate Care to RDs in Case of New Health Emergencies

The numerous lessons learned so far on health emergencies and RDs should represent the basis for the establishment of new healthcare policies and plans aimed at ensuring the preparedness of our healthcare systems in providing appropriate care to people living with RDs in case of eventual new emergencies. These policies and plans should benefit from the multi-stakeholder RDs networks (such as the ERNs), which should join forces at European, national, and local levels to minimise the economic, organisational, and health-related impacts and the negative effects of potential emergencies on the RDs community.

The implementation of new policies aimed at ensuring the care of RD patients could be ensured, besides the involvement of all RD actors, by a European approach that could develop a model framework that could then adapted and adopted in the single national healthcare systems.

Taking into consideration all the elements discussed above, a decalogue of organisational points to consider were developed as potential suggestions for the design of specific healthcare organisational plans aimed at ensuring appropriate care to people living with RDs in case of eventual future health emergencies [14]. Each of these organisational points, summarised in Table 1, could be considered as pragmatic fundamental principles for the design of joint actions or project proposals aimed at shaping and enforcing the healthcare systems toward a better care for RD patients.

## 6. Points to Consider

1. Assess the impact that the COVID-19 had on the rare diseases’ communities.

In order to have a clear picture of the different dimensions of rare diseases that were impacted by the pandemic, it would be absolutely essential to involve all the most relevant stakeholders in a deep and methodical analysis of every element (positive or negative) that occurred during the pandemic. This include the clinical and organisations issues emerged during the pandemic, the disruption of home and social care, the shortages of treatments and personal protective equipment, etc. The assessment of the COVID impact would provide precious information that can be used as a starting point to discuss any future actions aimed at ensuring the care and the management of rare diseases patients in the event of future pandemics.

2. Implement and adopt emergency plans aimed at ensuring the continuity of care both at organisational and clinical level for rare disease patients during health emergency.

Emergency plans should be designed or adopted to ensure that the diagnostic, monitoring, and therapeutic pathways of rare disease patients are still accessible for these patients. To do that, organisational and clinical procedures need to be delivered as soon as possible, defining the different healthcare services to be maintained and preserved in case of a new pandemic or other health emergencies. Thus, it is desirable that ad hoc organisational models are adopted at EU level to guarantee a homogeneous provision of care, considering also the geographical and cultural settings.

3. Develop/adopt national and local protocols for the protection/safeguard of people living with RDs during care delivery.

Disease-specific protocols on how to protect/safeguard RDs patients in the provision of care during a health emergency need to be implemented, such as the definition of protocols for social /physical distancing and other protective measures in in- and out-patient clinics and emergency rooms in case of an infectious-disease new health emergency.

4. Implement harmonised eHealth and telemedicine platforms for rare disease patients to limit the frequency of hospital face-to-face consultations.

The co-design of harmonised eHealth and telemedicine platforms dedicated to RDs patients and healthcare professionals is highly needed to be used not only during an eventual pandemic, but also in routinely care. Especially for RDs, in fact, this would imply considerable benefits in terms of economical and organisational burden both for the healthcare systems and for the patients and caregivers.

5. Ensure the availability of treatments and essential services for rare diseases (on and off-label).

RDs treatments, such as orphan drugs and off-label treatments, should be considered as essential medication to be ensured even during a health emergency. European and national policies could be optimised to ensure the accessibility of these drugs, especially when the active substance is not produced in Europe. In particular, a list of procedures and protocols should be designed to ensure the accessibility of these treatments, possibly with the support of all the stakeholders involved (national competent authorities, medicine agencies, pharma industries, patients representatives, RDs expert clinicians, and researchers, etc).

6. Ensure the prompt development of recommendations (or, if possible, Clinical Practice Guidelines) and communication material at different levels (European, national and local level) aimed at providing updated information for clinicians and patients on the potential correlation or impact of the health emergency on the different rare diseases/disease areas.

Building on existing national and European RDs networks, recommendations and reliable communication material should be produced promptly. In order to ensure that the material produced is user-friendly, a co-design could be organised and coordinated by existing RDs networks.

7. Collect, elaborate, and disseminate good practices related to the management of rare diseases during previous health emergencies and promote their application in appropriate contexts.

The recent global pandemic has encouraged several good practices in the RDs scenario. Those practices should be collected at national and European levels and be implemented as much as possible in the different contexts (healthcare providers, social care, patient organisations, etc.).

8. Implement the dissemination of reliable information on the health emergency and rare diseases at different levels (European, national and local level) using existing Networks.

The dissemination of reliable information should be arranged with the support of RDs European and national Networks, ensuring their full accessibility to the wider audience (RDs experts, patients, caregivers and the public), also in terms of language and readability.

9. Define essential social services, home care delivery, economical support etc. to be ensured during the emergency with dedicated protocols and resources.

A list of essential services to be delivered during a possible emergency should be defined and ensured for RDs patients. These include the provision of social services, home care delivery and home treatment plans and the definition of eventual preventive measures both for the patients and for the healthcare professionals delivering the services. The provision of home care delivery and home care treatment plans could represent a cost-effective solution to ensure the continuity of treatments and should be considered, whenever possible. The provision of economical support to RD patients should also be planned and considered, in particular for the most vulnerable patients and families.

10. Cross-border care services dedicated to RDs to be ensured by dedicated procedures during health emergency.

Even during a health emergency implying the impossibility of travelling, RDs patients needing to receive care far from their homes, should be able to travel and receive the care/treatment needed. It is also desirable to ensure that patients can access care or treatments prescribed by their treating specialist also in different healthcare settings that are closer to their homes. In order to achieve that, policies and strategic plans should be developed at European and national levels to ensure that RDs patients needing care/treatment abroad are enabled to travel despite the health emergency.

## 7. Conclusions

Healthcare emergencies such as COVID-19 have highlighted the strengths and weaknesses of the current RDs management. The knowledge and experience gained so far on this end need to be taken into account to ensure that our health systems will be ready to respond to the needs of people living with RDs in case of a possible future health emergencies. In order to implement those strategies, existing RDs networks need to join forces and act immediately on planning tangible initiatives that also consider the organisational and economic issues related to the care of those vulnerable patients.

## Figures and Tables

**Table 1:** Organisational points to consider in order to ensure appropriate care to RDs in case of new health emergencies.

Points to Consider	
1.	Assess the impact that the COVID-19 had on the rare diseases’ communities.
2.	Implement and adopt emergency plans aimed at ensuring the continuity of care both at organisational and clinical level for rare disease patients during health emergency.
3.	Develop/adopt national and local protocols for the protection/safeguard of people living with RDs during care delivery.
4.	Implement harmonised eHealth and telemedicine platforms for rare disease patients to limit the frequency of hospital face-to-face consultations.
5.	Ensure the availability of treatments and essential services for rare diseases (on and off-label).
6.	Ensure the prompt development of recommendations (or, if possible, Clinical Practice Guidelines) and communication material at different levels (European, national and local level) aimed at providing updated information for clinicians and patients on the potential correlation or impact of the health emergency on the different rare diseases/disease areas.
7.	Collect, elaborate and disseminate good practices related to the management of rare diseases during previous health emergencies and promote their application in appropriate contexts.
8.	Implement the dissemination of reliable information on the health emergency and rare diseases at different levels (European, national and local level) using existing Networks.
9.	Define essential social services, home care delivery, etc. to be ensured during the emergency with dedicated protocols and resources.
10.	Cross-border care services dedicated to RDs to be ensured by dedicated procedures during health emergency.

## References

[1] Nelson C., Lurie N., Wasserman J., Zakowski S. (2007). Conceptualizing and defining public health emergency preparedness. Am. J. Public Health.

[2] WHO Coronavirus Disease (COVID-19) Dashboard. https://covid19.who.int/.

[3] EURORDIS Rare Disease Patients’ Experience of COVID-19. https://www.eurordis.org/covid19survey.

[4] Ferrelli R.M., Gentile A.E., De Santis M., Taruscio D. (2017). Sustainable public health systems for rare diseases. Ann. Ist. Super. Sanita.

[5] De Santis M. (2019). Integrated care for healthcare sustainability for patients living with rare diseases. Ann. Ist. Super. Sanita.

[6] World Health Organisation COVID-19: Operational Guidance for Maintaining Essential Health Services during an Outbreak Interim Guidance, 25 March 2020. https://apps.who.int/iris/bitstream/handle/10665/331561/WHO-2019-nCoV-essential_health_services-2020.1-eng.pdf?sequence=1&isAllowed=y.

[7] Chung C.C., Wong W.H., Fung J.L., Hong Kong R.D., Chung B.H. (2020). Impact of COVID-19 pandemic on patients with rare disease in Hong Kong. Eur. J. Med. Genet..

[8] European Reference Networks. https://ec.europa.eu/health/ern_en.

[9] European Reference Networks and COVID-19. https://ec.europa.eu/health/ern/covid-19_en.

[10] What Are the ERNs Doing to Help Patients Affected by Rare Diseases and COVID-19?. https://ec.europa.eu/health/sites/health/files/ern/docs/covid19_erns_en.pdf.

[11] Share4Rare. https://www.share4rare.org.

[12] Italian National Center for Rare Diseases. https://www.iss.it/centro-nazionale-per-le-malattie-rare.

[13] Istituto Superiore di Sanità Covid-19 Report. https://www.iss.it/rapporti-iss-covid-19-in-english.

[14] Mazzoleni S., Turchetti G., Ambrosino N. (2020). The COVID-19 Outbreak: From “Black Swan” To Global Challenges and Opportunities. Pulmonology.

